# Reliability and validity of a General Nutrition Knowledge Questionnaire for adults in a Romanian population

**DOI:** 10.1038/s41430-020-0616-5

**Published:** 2020-03-31

**Authors:** Salomeia Putnoky, Ancuța Mioara Banu, Lavinia Cristina Moleriu, Sandra Putnoky, Denis Mihai Șerban, Mihai Dinu Niculescu, Costela Lăcrimioara Șerban

**Affiliations:** 1 Microbiology Department, “Victor Babes” University of Medicine and Pharmacy Timișoara, 16 Victor Babeș Blvd, 300226 Timișoara, Romania; 2 Maxilo-Facial Surgery Discipline, Faculty of Dental Medicine, “Victor Babes” University of Medicine and Pharmacy Timișoara, Bulevardul Take Ionescu 5, 300890 Timişoara, Romania; 3 Functional Sciences Department, “Victor Babes” University of Medicine and Pharmacy Timișoara, 14 Spl. Tudor Vladimirescu, 300172 Timişoara, Romania; 4 “Victor Babes” University of Medicine and Pharmacy Timișoara, Eftimie Murgu Square Nr 4, 300041 Timișoara, Romania; 5 Psychiatric Clinic, Emergency Clinical County Hospital, 21 Iancu Văcărescu Str, 300425 Timișoara, Romania; 6 Obstetrics and Gynecology Department, “Victor Babes” University of Medicine and Pharmacy Timișoara, 1-3 Al. Odobescu Str, 300202 Timișoara, Romania; 7 Advanced Nutrigenomics, 130 Rainbow Ct, Cary, NC 27511 USA; 8 Genetics Compartment, Emergency Hospital for Children Louis Ţurcanu, 2 Doctor Iosif Nemoianu Str, 300011 Timișoara, Romania

**Keywords:** Epidemiology, Nutrition

## Abstract

**Background:**

Nutritional knowledge assessment is an important component in nutrition research, and a prerequisite for the implementation of many policies and programs aimed at improving eating behavior. In order to generate objective results, validated tools for a given population must be employed. The aim of this study was to determine the validity and reliability of a nutrition knowledge questionnaire for Romanian adults.

**Methods:**

Kleimann’s version of a General Nutrition Knowledge Questionnaire, was translated and adapted to Romanian language, culture, and cuisine. The final format was developed in several steps and used four components: internal and external reliability were assessed in a general population sample (n1 = 412), respectively in a subgroup (n2 = 46) from Component 1; Component 3 assessed construct validity (n3 = 96) using the “known-groups” method; Component 4 (convergent validity, n4 = 508) tested the association between socio-demographic characteristics and nutrition knowledge.

**Results:**

The overall internal reliability was 0.878 and the external reliability was >0.880 in all sections, and overall. Specialists had higher scores than nonspecialists, with a very large effect size. In the general population, females scored higher than males, and middle-aged and older adults scored higher than young adults. Higher scores were associated with higher levels of education. The characteristics of individuals prone to giving wrong answers were: males (beta = 0.170), high school or less (beta = 0.167), and no training in nutrition (beta = 0.154).

**Conclusions:**

The Romanian version of the General Nutrition Knowledge Questionnaire is a reliable and valid tool for measuring nutrition knowledge in adults.

## Introduction

There is a very young history for nutrition and dietetics professionals in Romania, since this profession was first recognized only in 2015 [1], and the application norms of the Law of Dietitian were published early in 2019 [2]. Until the legal recognition of the profession of dietitian, the role of nutritional management related to food choices, dieting, and special diets for medical conditions, was performed by medical doctors in different medical specialties such as family doctors or specialists in diabetes, nutrition, and metabolic diseases [3].

In several studies on different populations, it was shown that higher nutrition knowledge was associated with an adoption of healthier lifestyles and food choices [4, 5], albeit this association is still controversial or has limited value [6]. Other factors, including social-economic status of the family and traditions, taste preferences, and genetics, are strong drivers that influence individual dietary habits [6–8]. Previous research indicated that the food choices of Romanians are largely driven by economic reasons [9].

Currently, there are no validated tools to measure nutrition knowledge of the general adult public in Romania. The purpose of this study was to determine the validity and reliability of a General Nutrition Knowledge Questionnaire in Romanian population, in order for it to be further used in subsequent research.

## Materials and methods

**Fig. 1:**
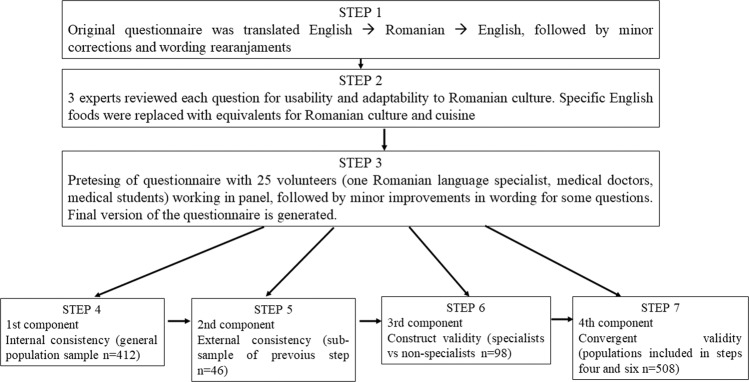
Flow diagram for the steps, components, and populations used in reliability and validity testing of General Nutrition Knowledge Questionnaire for adults in a Romanian population.

The most known questionnaire of nutrition knowledge is Parmenter’s work [10], developed in 1990s and validated in English, and which is used as starting point for generating nutrition knowledge questionnaires in different languages [11–14] or for different purposes [15–17], ever since it was published. More recently, Kliemann et al. [18] updated the version developed by Parmenter and Wandle with the latest expert recommendations. For our purposes we have used the version updated by Kliemann in the following steps (Fig. 1):

- Step 1. The original questionnaire was translated to Romanian and then back to English by two independent translators. The English original version was compared with the backward translated version, with corresponding minor corrections being made in the Romanian version.
- Step 2. A panel of three experts, with medical background and expertise in human nutrition and dietetics, nutritional epidemiology, and nutrigenomics/nutrigenetics, critically reviewed each question from the Kliemann version for usability and adaptability to Romanian culture. Following this step, all questions were kept but some specific English foods were replaced with foods known and utilized in Romania.
- Step 3. Pretesting of questionnaire was performed on 25 volunteers, working in panel. The main point of pretesting was to provide a clear and easy understanding for each question. The outcome of the pretesting panel consisted in minor improvements in the wording of some questions.
- Step 4. A general population sample of 412 adults was used for determining the internal reliability of the questionnaire per each section and per total (Component 1).
- Step 5. External reliability of the questionnaire was assessed with a subgroup of 46 adults from general sample population, who were retested after at least 30 days after the first round (Component 2).
- Step 6. Construct validity was assessed with 48 students/specialists in dietetics (last year undergraduates, master students, and recent—up to 3 years—master graduates) and with 48 mathematics/informatics undergraduate students, using the “known-groups” method (Component 3). The sample size was calculated in order to assure a large effect size, with a power of at least 80%, using one-sided tests.
- Step 7. Combined participants in steps 4 and 6 were used for the assessment of convergent validity that explored associations between nutrition and dietetics knowledge and social and demographic characteristics **(**Component 4). The sample size provided by this component is ensuring a power of at least 80%, with a margin of error of 5%.

The questionnaire contained, besides specific nutritional knowledge questions, demographic questions such as: gender, self-evaluation of health status (with five possible ordinal categories from excellent to poor), marital status (with six possible nominal categories), number of children (ordinal with five categories, from zero to four or more children), presence of minors in household (dichotomic, yes/no), highest education level (ordinal, with eight possible answers from gymnasium school or less to PhD), if they had training in nutrition (dichotomic, yes/no), and a self-evaluation of nutritional status by self-reported weight and height.

### Ethics approval

The Institutional Review Board at the Victor Babes University of Medicine and Pharmacy Timisoara, Romania approved this research protocol. All subjects included in the study provided informed consent before participation.

### Data management and data analysis

Up to step 4 the paper-based version of the questionnaire was used. For the following steps an online questionnaire was employed. Since the possibility to enter bias by using the internet or communicate with others in order to get the right answers was big if posted freely online, the researchers invited individuals or groups, and opened the questionnaire then closed it after the completion was done. At least one member of the research team was always present in order to limit the access to sources of information and communication between participants, if approached in groups.

Databases were exported and IBM-SPSS version 21 was used to process the data. Scores for questions were calculated in two ways [17]:

- Type A scoring system: for each correct answer one point was added. For wrong answers or if the responder chose “I do not know” option, no points were added.
- Type B scoring system: for each correct answer one point was added, but for wrong answers one point was subtracted. If the responder chose “I do not know” option, no points were added.

Type B of score computing was used for Component 4, while Type A was used in Components 2, 3, and 4.

For each section and for the entire questionnaire the achievement score was computed, taking into account the score obtained by each scoring system and the maximum score that could be obtained by sections and for the entire questionnaire. For the fourth component, the difference between type A and type B scoring was computed for the purpose of identifying those individuals who gave wrong answers. For demographical questions, some categories with few responses were collapsed, and in order to facilitate the better understanding of the meaning of such questions.

Body mass index was calculated using self-reported height and weight, and participants further classified into normal or overweight and obese.

Continuous data are presented as mean ± standard deviation or median (interquartile range (IQR)). Categorical data are presented as percentages. For comparison of proportions chi-square test was used. For comparing parametric data on a 2-category factor, *t*-test was used with appropriate decision for equality of variances with Levene’s test, and when comparing nonparametric or ordinal data, the Mann–Whitney test was used. For three or more groups with data that did not assume normality, Kruskal–Wallis test was the choice. Bonferroni correction was applied for several comparisons following significant Kruskal–Wallis test. Paired comparisons between two different scoring systems were assessed with Wilcoxon signed-ranks test. A linear regression model was built, with the difference between type A and type B scoring system as a dependent variable, and demographic factors as independent variables.

## Results

### Internal and external reliability

Internal reliability, which measures the degree of correlation between different items of the questionnaire, was assessed on a general population sample (*n* = 412) by Cronbach’s alfa. Per the entire questionnaire, it had an overall alpha value of 0.879, larger than the threshold of 0.7. Sections 1 and 3 had an individual alfa lower than the threshold, and sections 2 and 4 had larger Cronbach’s alfa values than the threshold (Table 1).

**Table 1:** Internal reliability for current study (Component 1, *n*1 = 412) and comparison with the results of questionnaires developed in other countries.

	Items in Romanian questionnaire (current study)	Romanian sample (current study) *n*_1_ = 412	UK sample (*n* = 168) [10]	Australian sample (*n* = 156) [19]	Turkish sample (*n* = 195) [13]	Uganda sample (*n* = 117) [12]	UK sample (*n* = 266) [18]	Japanese sample (*n* = 1182) [11]
Overall	88	0.88	0.97	0.92	0.89	0.95	0.93	0.95
Section 1 Expert recommendations	18	0.53	0.70	0.53	0.47	0.56	0.70	0.78
Section 2 Food groups	36	0.82	0.95	0.88	0.88	0.85	0.86	0.94
Section 3 Healthy food choices	13	0.53	0.76	0.55	0.43	0.85	0.72	0.31
Section 4 Diet, disease and weight associations	21	0.72	0.94	0.73	0.81	0.91	0.77	0.81

External consistency (or test–retest) was assessed on a subgroup of the population sample (*n* = 46). The interclass correlation coefficient was higher than 0.85 for all sections and overall (Table 2).

**Table 2:** External reliability (Interclass correlation coefficient) for current study (Component 2) and comparison with the results of questionnaires developed in other countries.

	Items in Romanian questionnaire (current study)	Romanian sample (current study) *n*_2_ = 46	UK sample (*n* = 168) [10]	Australian sample (*n* = 156) [19]	Turkish sample (*n* = 195) [13]	Uganda sample (*n* = 117) [12]	UK sample (*n* = 266) [18]	Japanese sample (*n* = 1182) [11]
Overall	88	0.96	0.98	0.87	0.86	0.89	0.93	0.75
Section 1 Expert recommendations	18	0.94	0.80	0.37	0.56	0.80	0.70	0.67
Section 2 Food groups	36	0.96	0.94	0.85	0.85	0.77	0.86	0.67
Section 3 Healthy food choices	13	0.88	0.87	0.75	0.57	0.84	0.72	0.44
Section 4 Diet, disease, and weight associations	21	0.86	0.97	0.74	0.60	0.80	0.77	0.68

### Assessment of construct validity

For the construct validity, the two groups considered further (specialists and nonspecialists) were equal in size, *n* = 48/group. The distribution of gender was similar, but specialists who had a higher age, a lower BMI, were more likely to be in a relationship and have children, and considered to have a better health status as compared with nonspecialists (Table 3).

**Table 3:** Demographic information by categories of responders (Component 3) *n*_3_ = 96.

	Categories		Significance
	Nonspecialists (48)	Specialists (48)	
Gender			
M	10 (20.8%)	13 (27.1%)	0.743^a^
F	38 (79.2%)	35 (72.9%)	
Health status			
Poor	0 (0.0%)	1 (2.1%)	0.029^b^
Fair	17 (35.4%)	5 (10.4%)	
Good	16 (33.3%)	21 (43.8%)	
Very good	13 (27.1%)	16 (33.3%)	
Excellent	2 (4.2%)	5 (10.4%)	
Marital status			
Alone	39 (81.3%)	17 (27.1%)	<0.001^a^
With partner	9 (2.1%)	31 (50.0%)	
Children			
No	47 (97.9%)	32 (66.7%)	<0.001^a^
Yes	1 (2.1%)	16 (33.4%)	
Age			
Mean (±SD) Median (IQR)	22.0 ± 3.7 21.0 (1.8)	33.7 ± 9.7 32.5 (15.8)	<0.001^c^
Body mass index (BMI)			
Mean (±SD) Median (IQR)	23.5 ± 4.1 22.4 (6.2)	22.7 ± 3.4 22.2 (4.9)	0.420^b^

^a^Chi-square test.

^b^Mann–Whitney test.

^c^*t*-test.

Mean ± SD and medians (IQR) per sections and per total in categories of specialists and nonspecialists are presented in Table 4. With a very large effect size, the overall score and per sections scores were significantly higher for specialists when compared with nonspecialists (Table 4).

**Table 4:** Sections scores and total score for evaluation of specialists and nonspecialists (Component 3).

	Categories			
	Nonspecialists (*n* = 48)	Specialists (*n* = 48)	Significance	Size effect (Cohen’s D)
Section 1 (max 18 points) Expert recommendations	10.6 ± 1.9 11 (3)	13.9 ± 2.0 14 (3)	<0.001^a^	1.68
Section 2 (max 36 points) Food groups	18.6 ± 4.6 19 (3)	28.3 ± 3.4 28 (5)	<0.001^a^	3.39
Section 3 (max 13 points) Healthy food choices	8.3 ± 2.1 8 (3)	10.6 ± 1.7 11 (2)	<0.001^a^	1.18
Section 4 (max 21 points) Diet, disease, and weight associations	12.1 ± 3.2 12.5 (5)	17.2 ± 2.5 18 (3)	<0.001^a^	1.77
Total score (max 88 points)	49.7 ± 9.6 49.5 (14.75)	70.0 ± 7.3 71.0 (9)	<0.001^a^	2.38

Data in table represent mean ± SD and median (IQR).

^a^*t*-test.

### Assessment of convergent validity

Table 5 presented the knowledge scores per sections in a sample of 508 responders by different demographic characteristics of the sample, using two different methods of score calculation, with a total possible score of 88 points. Type B scoring had significant lower median achievement scores in all sections and for the entire questionnaire, as compared with type A (*p* < 0.001).

**Table 5:** Sections scores and total score for evaluation of general population (Component 4) *n*_4_ = 508 participants.

	Type A scoring system: correct answer 1 point, else 0 points					Type B scoring system: correct answer 1 point, wrong answer −1 points and “I am not sure” option 0 points				
	Section 1 Max 18	Section 2 Max 36	Section 3 Max 13	Section 4 Max 21	Total Max 88 points	Section 1 Max 18	Section 2 Max 36	Section 3 Max 13	Section 4 Max 21	Total Max 88 points
All sample										
	12.0 (3.75)	23.0 (7)	10.0 (3)	15.0 (4)	58.0 (17)	7.0 (6)	13.0 (10)	7.0 (5.75)	10.0 (8)	36.0 (22)
Median achievement	66.7%	63.8%	76.9%	71.4%	65.9%	38.8%	36.1%	53.8%	47.6%	40.9%
Gender										
M (138)	12.0 (4)^a^	23.0 (8)^a^	9.0 (4)^a^	14.0 (6)^a^	56.0 (16)^a^	7.0 (6)^a^	12.0 (11)^a^	5.0 (7)^a^	9.0 (7.5)^a^	33.0 (25)^a^
F (353)	12.0 (3)^a^	23.0 (7)^a^	10.0 (3)^b^	15.0 (5)^a^	59.0 (16)^a^	7.0 (6)^a^	13.0 (10)^a^	7.0 (4)^b^	10.0 (6)^b^	37.0 (20)^b^
Age categories**										
<30 (199)	11.0 (4)^a^	21.0 (7)^a^	9.0 (3.75)^a^	14.0 (6)^a^	55.0 (17)^a^	6.0 (4)^a^	11.0 (10)^a^	6.0 (6)^a^	9.0 (9)^a^	32.0 (20.75)^a^
30–49 (192)	12.0 (4)^b^	24.0 (7)^b^	10.0 (3)^b^	15.0 (4)^a^	61.0 (14)^b^	7.0 (5)^a^	15.0 (10)^b^	7.0 (6)^a^	10.0 (6)^a^	38.0 (21)^b^
≥50 (100)	12.0 (4)^b^	24.0 (6.5)^b^	10.0 (3)^a,b^	15.0 (5)^a^	60.0 (15)^b^	8.0 (5)^b,a^	15.0 (10)^b^	7.0 (6)^a^	11.0 (8)^a^	39.0 (24.5)^b^
BMI categories**										
Normal weight (315)	12.0 (4)^a^	23.0 (7)^a^	10.0 (3)^a^	15.0 (6)^a^	58.0 (17)^a^	7.0 (6)^a^	13.0 (11)^a^	7.0 (5)^a^	10.0 (8)^a^	36.0 (22)^a^
Overweight (131)	12.0 (4)^a^	23.0 (6)^a^	10.0 (3)^a^	14.0 (4)^a^	58.0 (15)^a^	8.0 (5)^a^	13.0 (10)^a^	7.0 (6)^a^	10.0 (8)^a^	36.0 (21)^a^
Obese (62)	12.0 (4)^a^	21.5 (7)^a^	10.0 (3)^a^	14.0 (4)^a^	57.0 (14.5)^a^	7.0 (7)^a^	11.0 (10)^a^	7.0 (4)^a^	10.0 (7)^a^	36.0 (22.25)^a^
Excellent or very good health status*										
No (371)	12.0 (3)^a^	22.0 (7)^a^	9.0 (3.5)^a^	15.0 (4)^a^	58.0 (15)^a^	7.0 (5)^a^	12.0 (10)^a^	7.0 (6)^a^	10.0 (8)^a^	36.0 (22)^a^
Yes (120)	12.0 (4)^a^	24.0 (7)^b^	10.0 (3)^a^	15.0 (6)^a^	61.0 (19)^b^	7.0 (5)^a^	14.0 (13)^b^	7.0 (4)^a^	10.0 (9)^a^	37.0 (25)^a^
Marital status*										
Alone or without a relation (228)	11.0 (14)^a^	22.0 (8)^a^	9.0 (4)^a^	14.0 (5)^a^	57.0 (16.5)^a^	6.0 (5)^a^	12.0 (11)^a^	6.0 (6)^a^	9.0 (8)^a^	34.0 (21)^a^
Married or in a relation (363)	12.0 (14)^b^	23.0 (6)^b^	10.0 (3)^b^	15.0 (5)^a^	60.0 (15)^b^	8.0 (5)^b^	14.0 (10)^b^	7.0 (5)^b^	10.0 (7)^a^	38.0 (24)^b^
Children *										
No children (297)	12.0 (3)^a^	22.0 (8)^a^	9.0 (3)^a^	14.0 (5)^a^	57.0 (18)^a^	6.0 (5)^a^	12.0 (10)^a^	7.0 (6)^a^	9.0 (8)^a^	34.0 (22.75)^a^
At least one (211)	12.0 (4)^a^	24.0 (6)^b^	10.0 (3)^a^	15.0 (4)^a^	60.0 (13.75)^b^	8.0 (5)^b^	15.0 (10)^b^	7.0 (5) ^a^	10.0 (7)^a^	38.0 (21.75)^b^
Living with minors*										
No (370)	12.0 (3.75)^a^	22.0 (8)^a^	9.0 (4)^a^	14.0 (5)^a^	58.0 (17.5)^a^	7.0 (6)^a^	12.0 (10)^a^	7.0 (6)^a^	9.0 (8)^a^	35.0 (22)^a^
Yes (121)	12.0 (3.75)^a^	24.0 (5)^b^	10.0 (3)^a^	15.0 (4)^a^	60.5 (12.75)^b^	8.0 (5)^a^	15.0 (9)^b^	7.0 (4)^a^	11.0 (6.75)^b^	38.5 (21)^b^
Education*										
High school or less (190)	11.0 (4)^a^	21.0 (7)^a^	9.0 (4)^a^	13.0 (6)^a^	54.5 (17)^a^	6.0 (5.25)^a^	10.0 (8)^a^	6.0 (6)^a^	7.0 (8)^a^	29.0 (23)^a^
At least college degree (318)	12.0 (4)^b^	24.0 (7)^b^	10.0 (3)^b^	15.0 (5)^b^	60.5 (14)^b^	8.0 (5)^b^	15.0 (11)^b^	7.0 (4)^b^	11.0 (7)^b^	39.0 (22)^b^

Numbers in tables represent median (IQR). Values with different letters in superscript denote statistical significance.

*Mann–Whitney test. **Kruskal–Wallis test with Bonferroni correction for multiple comparisons.

### Demographic predictors of choosing the wrong answer

Table 6 contains demographic significant predictors of high difference between the two scoring systems, when controlling for age, BMI, marital status, health perception, and the presence of children. Males (2.6 times more likely), lower level of education (2.3 times more likely), and no previous studies in nutrition (3.2 times more likely) were the independent variables that contributed significantly to the model.

**Table 6:** Prediction of the difference of two scoring systems from demographic factors.

Model		Unstandardized coefficients		Standardized coefficients	95.0% confidence interval for B	
		*B*	Std. error	Beta	Lower bound	Upper bound
1	(Constant)	28.880	1.439		26.053	31.707
	Gender (females)	−2.604	0.672	−0.170	−3.924	−1.283
	Education level (at least college)	−2.378	0.654	−0.167	−3.662	−1.093
	Studies in nutrition (yes)	−3.216	0.908	−0.154	−5.001	−1.431

Dependent variable: difference of two scoring system.

Independent variables: Gender, age categories, BMI categories, marital status, health perception, presence of children, level of education, and studies in nutrition.

*F*(8499) = 7.95, *p* < 0.001, adjusted *R* square = 0.099.

## Discussion

Since currently there is no validated tool to collect general nutrition knowledge in Romanian adults, the present study aimed to validate and adapt to Romanian language the General Nutrition Knowledge Questionnaire updated recently by Kliemann et al. [18] and developed by Parmenter and Wardle [10], which is exploring general nutrition knowledge with the following sections: dietary recommendation; food groups; healthy food choices; and diet, disease, and weight associations. The original version of the questionnaire is in English, but besides translation, in Romanian version, less common dishes/foods were replaced with similar, more common foods present in Romanian eating pattern/cuisine.

The results from sections and for the entire questionnaire were used to assess overall internal reliability, external reliability, construct validity, and convergent validity of the questionnaire (Tables 1–4) These criteria were adequate and comparable to other published studies, as presented in Tables 1 and 2 [11, 13, 18–22]. Lower internal reliability for sections 1 (dietary recommendations) and 3 (healthy food choices) were explained by others due to the high heterogeneity in background of responders and possibly by different interpretations of the nutritional recommendations in the absence of adequate training [12, 23].

The scores performed by specialists who had several training courses in nutrition were, with a very large effect size, significantly higher than those of nonspecialists, who did not have training in nutrition (Table 3), and were comparable to other similar studies [10, 11, 13, 18]. These results validated the construct validity.

Overall achievement score was low, with its median at 65.9% (Table 4). Lower scores were reported for dietary recommendations (median A vs. B scoring system 66.7% vs. 38,8%) and food groups (median A vs. B scoring system 63.8% vs. 36.1%). Higher scores were reported for healthy food choices (median A vs. B scoring system 76.9% vs. 53,8%), disease and weight associations with diet (median A vs. B scoring system 71.4% vs. 47,6%), and overall achievement scores of 65.9% and 40.9% in type A, respective type B scoring system. In a previous study performed in the 90s in England, Parmenter had found much lower scores in nutrition knowledge than the maximum that could be achieved, when using the tool in a general population [24]. Our results indicated low theoretical background in the Romanian adult population.

Previous studies [10, 19, 25, 26] have reported that women tend to have higher knowledge scores as compared with men. In the sample analyzed in the fourth component (Table 4), women had more precision in nutrition knowledge and therefore had higher scores only in type B scoring system, as compared with men.

Significant differences were assessed in our sample between middle-aged adults and seniors, when compared with young adults. Higher knowledge in middle-aged and older adults could be a result of life experience (Table 4). Other recent studies performed in countries with better nutrition curricula in schools showed similarity in knowledge between young adults who benefited from a good nutrition curricula, and the older adults (due to their life experience) [18].

In our study, the higher education status was associated with better scores in nutrition knowledge, as already indicated previously [24, 25]. Non-single status, the presence of children in families, and the presence of minors in families were items associated with higher knowledge, since the presence of a child often raises awareness about healthy lifestyles, including nutrition [24, 27, 28].

Although each question had the option “not sure” and responders were advised to choose that option over guessing, several classes of responders chose to force an answer and for some, the answer was incorrect. The two scoring systems assessed different aspects of the nutrition knowledge: if type A was a positive score, type B scoring was focused on wrong answers. Using their difference, we were able to identify and quantify those individuals who were more likely to give wrong answers. Demographic predictors of giving a wrong answer were male gender, high school or lower education, and no studies in nutrition. These data support the results of univariate analysis and previously published studies [11, 18, 24, 29, 30].

When comparing the present study with other similar studies (Tables 1 and 2), internal and external reliability coefficients for each section were, in general, similar. When compared with other studies, the present study had the highest correlation coefficients for external reliability sections 1–3 (Table 2), while having the lowest coefficients for internal reliability sections 2, 4, and overall (Table 1).

This study has some limitations. First of all, it is already known [31] that selection bias could exist for individuals willing to participate in a questionnaire targeting nutrition knowledge. These individuals were more likely to be educated, to have a genuine interest in healthy lifestyle and to have a better health status. The income was not assessed, and therefore no generalization can be made over different economical strata. Another limitation consists in the geographical and cultural representation of the selected participants (western Romania).

The Romanian version of Nutrition Knowledge Questionnaire is the first validated tool designed to collect general nutrition knowledge in Romanian adults, to the best of our knowledge. The questionnaire overall, and its sections, had adequate construct and convergent validity, internal reliability, and external reliability, which make it a valuable tool in assessing nutritional knowledge.

## Supplementary information

General Nutrition Knowledge Questionnaire in Romanian

Kliemann's General Nutrition Knowledge Questionnaire (18)
