# Supplementary material for: Reliability and validity of a General Nutrition Knowledge Questionnaire for adults in a Romanian population
Source: Eur J Clin Nutr. 2020 Mar 31;74(11):1576–84. doi: 10.1038/s41430-020-0616-5 (PMC7606136; doi:10.1038/s41430-020-0616-5)
Supplement: Supplementary file 1 — General Nutrition Knowledge Questionnaire in Romanian [file 41430_2020_616_MOESM1_ESM.docx]

| **CHESTIONAR DE CULTURĂ GENERALĂ A NUTRIȚIEI** | | | | | | | | | | |
| --- | --- | --- | --- | --- | --- | --- | --- | --- | --- | --- |
|  | | | | | | | | | | |
| \| **Acesta este un sondaj, nu un test. Răspunsurile dumneavoastră ne vor ajuta să identificăm ce sfaturi alimentare oamenii le găsesc confuze. Este important să îl completați de unul singur. Răspunsurile dumneavoastră vor rămâne anonime. Dacă nu știți răspunsul, marcați "nu sunt sigur" decât să răspundeți la întâmplare.**  **Mulțumim pentru timpul acordat!** \| \| --- \| | | | | | | | | | | |
|  | | | | | | | | | | |
| **Secțiunea 1: Primele întrebări sunt despre sfaturile pe care considerați că le oferă experții.** | | | | | | | | | | |
|  | | | | | | | | | | |
| 1. | Experții în domeniul sănătății recomandă să se consume mai mult, aceeași cantitate sau mai puțin din următoarele alimente? (bifați o casetă pentru fiecare aliment) | | | | | | | | | |
|  | | Mai mult | | Aceeași | | Mai puțin | | Nu sunt sigur |  |  |
| Fructe | | ✓ | | □ | | □ | | □ |  |  |
| Alimente și băuturi cu zahăr adăugat | | □ | | □ | | ✓ | | □ |  |  |
| Legume | | ✓ | | □ | | □ | | □ |  |  |
| Alimente grase | | □ | | □ | | ✓ | | □ |  |  |
| Carne roșie procesată | | □ | | □ | | ✓ | | □ |  |  |
| Cereale integrale | | ✓ | | □ | | □ | | □ |  |  |
| Alimente sărate | | □ | | □ | | ✓ | | □ |  |  |
| Apă | | ✓ | | □ | | □ | | □ |  |  |
|  | | | | | | | | | | |
| 2. | Câte porții de fructe și legume recomandă experții să se consume într-o zi? (O porție este, de exemplu, un măr sau un pumn de morcovi tăiați) (bifați o casetă) | | | | | | | | | |
| 2 | | □ | | |  | | | | | |
| 3 | | □ | | |  | | | | | |
| 4 | | □ | | |  | | | | | |
| 5 sau mai mult | | ✓ | | |  | | | | | |
| Nu sunt sigur | | □ | | |  | | | | | |
|  | | | | | | | | | | |
| 3. | Ce tipuri de grăsimi recomandă experții să se consume mai puțin?  (bifați o casetă pentru fiecare aliment) | | | | | | | | | |
|  | | Consumă mai puțin | Nu consuma mai puțin | | | Nu sunt sigur |  | | | |
| Grăsimi nesaturate | | □ | ✓ | | | □ |  | | | |
| Grăsimi trans | | ✓ | □ | | | □ |  | | | |
| Grăsimi saturate | | ✓ | □ | | | □ |  | | | |
|  | | | | | | | | | | |
| 4. | Ce tipuri de lactate recomandă experții să fie consumate? (bifați o casetă) | | | | | | | | | |
| Lactate cu grăsimi integrale (de exemplu, lapte integral, lapte gras) | | □ |  | | | | | | | |
| Lactate cu grăsimi reduse(e.g. lapte degresat sau semi-degresat) | | ✓ |  | | | | | | | |
| Ambele tipuri de lactate, degresate și cu grăsimi integrale | | □ |  | | | | | | | |
| Niciuna, lactatele trebuie evitate | | □ |  | | | | | | | |
| Nu sunt sigur | | □ |  | | | | | | | |

|  | | | |
| --- | --- | --- | --- |
| 5. | De câte ori pe săptămână recomandă experții să se consume pește gras (de exemplu, somon, macrou)? (bifați o casetă) | | |
| 1-2 ori pe săptămână | | ✓ |  |
| 3-4 ori pe săptămână | | □ |  |
| În fiecare zi | | □ |  |
| Nu sunt sigur | | □ |  |
|  | | | |
| 6. | Care estenumărul maxim de porții de băuturi alcoolice acceptate a fi consumate zilnic? 1 porție = 30-40 ml tărie cu 40% alcool , de exemplu un păhăruț de țuică, un shot de vodcă; 330 ml de bere 5%, adică o doză mică; 150 ml vin 12%, adică jumătate de pahar de vin (bifați o casetă) | | |
| 1 porție, atât pentru bărbați, cât și pentru femei | | □ |  |
| 2 porții, atât pentru bărbați, cât și pentru femei | | □ |  |
| 2 porții pentru bărbați și 1 porție pentru femei | | ✓ |  |
| 3 porții pentru bărbați și 2 porții pentru femei | | □ |  |
| Nu sunt sigur | | □ |  |
|  | | | |
| 7. | De câte ori pe săptămână recomandă experții să se consume micul dejun? (bifați o casetă) | | |
| 3 ori pe săptămână | | □ |  |
| 4 ori pe săptămână | | □ |  |
| În fiecare zi | | ✓ |  |
| Nu sunt sigur | | □ |  |
|  | | | |
| 8. | Câte porții de fructe și legume se consideră că a consumat o persoană care a băut două pahare (1 pahar = 200ml) de suc de fructe într-o zi? (bifați o casetă) | | |
| Niciuna | | □ |  |
| O porție | | ✓ |  |
| Două porții | | □ |  |
| Trei porții | | □ |  |
| Nu sunt sigur | | □ |  |
|  | | | |
| 9. | Cât de mult din dieta unei persoane ar trebui să fie alimentele cu conținut de amidon?(bifați o casetă) | | |
| Un sfert | | □ |  |
| O treime | | ✓ |  |
| Jumătate | | □ |  |
| Nu sunt sigur | | □ |  |

| **Secțiunea 2: Experții clasifică alimentele în grupe. Noi suntem interesați să vedem dacă oamenii cunosc grupele alimentare și nutrienții pe care îi conțin.** | | | | | | | | | | | | | | |
| --- | --- | --- | --- | --- | --- | --- | --- | --- | --- | --- | --- | --- | --- | --- |
|  |  | | | | | | | | | | | | | |
| 1. | Credeți că aceste alimente și băuturi sunt bogate sau sărace în zahăr adăugat?  (bifați o casetăpentru fiecare aliment) | | | | | | | | | | | | | |
|  | | | | | | Bogate în zahăr adăugat | | Sărace în zahăr adăugat | | | Nu sunt sigur | |  | |
| Băuturi tip cola dietetice | | | | | | □ | | ✓ | | | □ | |  | |
| Iaurt natural | | | | | | □ | | ✓ | | | □ | |  | |
| Înghețată | | | | | | ✓ | | □ | | | □ | |  | |
| Ketchup de roșii | | | | | | ✓ | | □ | | | □ | |  | |
| Pepene | | | | | | □ | | ✓ | | | □ | |  | |
|  | | | | | | | | | | | | | | |
| 2. | | | | Credeți că aceste alimente sunt bogate sau sărace în sare? (bifați o casetăpentru fiecare aliment) | | | | | | | | | | |
|  | | | | | | Bogate în sare | | Sărace în sare | | | Nu sunt sigur | |  | |
| Cereale pentru micul dejun | | | | | | ✓ | | □ | | | □ | |  | |
| Legume congelate | | | | | | □ | | ✓ | | | □ | |  | |
| Pâine | | | | | | ✓ | | □ | | | □ | |  | |
| Iahnie de fasole | | | | | | ✓ | | □ | | | □ | |  | |
| Carne roșie | | | | | | □ | | ✓ | | | □ | |  | |
| Supă la conservă sau la plic | | | | | | ✓ | | □ | | | □ | |  | |
|  | | | | | | | | | | | | | | |
| 3. | | | | Credeți că aceste alimente sunt bogate sau sărace în fibre? (bifați o casetă box pentru fiecare aliment) | | | | | | | | | | |
|  | | | | | | Bogate în fibre | | Sărace în fibre | | Nu sunt sigur | | |  | |
| Ovăz | | | | | | ✓ | | □ | | □ | | |  | |
| Banane | | | | | | ✓ | | □ | | □ | | |  | |
| Orez alb | | | | | | □ | | ✓ | | □ | | |  | |
| Ouă | | | | | | □ | | ✓ | | □ | | |  | |
| Morcovi | | | | | | ✓ | | □ | | □ | | |  | |
| Paste | | | | | | □ | | ✓ | | □ | | |  | |
|  | | | | | | | | | | | | | | |
| 4. | | | | Credeți că aceste alimente sunt o bună sursă de proteine? (bifați o casetă pentru fiecare aliment) | | | | | | | | | | |
|  | | | | | | Sursă bună de proteine | | Sursă slabă de proteine | | | Nu sunt sigur | |  | |
| Pui | | | | | | ✓ | | □ | | | □ | |  | |
| Brânză | | | | | | ✓ | | □ | | | □ | |  | |
| Fructe | | | | | | □ | | ✓ | | | □ | |  | |
| **Fasole boabe** | | | | | | ✓ | | □ | | | □ | |  | |
| Unt | | | | | | □ | | ✓ | | | □ | |  | |
| Nuci | | | | | | ✓ | | □ | | | □ | |  | |
| 5. | | | Care din următoarele alimente sunt considerate de experți alimente cu conținut de amidon? (bifați o casetă box pentru fiecare aliment) | | | | | | | | | | | |
|  | | | | | | | Aliment cu amidon | Aliment fără amidon | | | Nu sunt sigur | |  | |
| Brânză | | | | | | | □ | ✓ | | | □ | |  | |
| Paste | | | | | | | ✓ | □ | | | □ | |  | |
| Cartofi | | | | | | | ✓ | □ | | | □ | |  | |
| Nuci | | | | | | | □ | ✓ | | | □ | |  | |
| Banane verzi | | | | | | | ✓ | □ | | | □ | |  | |
|  | |  | | | | | | | | | | | | |
| 6. | | Ce tip de grăsime se găsește, în principal, în următoarele alimente? (bifați o casetă pentru fiecare aliment) | | | | | | | | | | | | |
|  | | | | | Grăsimi polinesaturate | Grăsimi mononesa-turate | | | Grăsimi nesaturate | | | Colesterol | | Nu sunt sigur |
| Ulei de măsline | | | | | □ | ✓ | | | □ | | | □ | | □ |
| Unt | | | | | □ | □ | | | ✓ | | | □ | | □ |
| Ulei de floarea soarelui | | | | | ✓ | □ | | | □ | | | □ | | □ |
| Ouă | | | | | □ | □ | | | □ | | | ✓ | | □ |
|  | | | | | | | | | | | | | | |
| 7. | | | Care dintre aceste alimente are cele mai multe grăsimi trans? (bifați o casetă) | | | | | | | | | | | |
| Biscuiți, prăjituriși produse de patiserie | | | | | | ✓ | |  | | | | | | |
| Pește | | | | | | □ | |  | | | | | | |
| Ulei de ricin | | | | | | □ | |  | | | | | | |
| Ouă | | | | | | □ | |  | | | | | | |
| Nu sunt sigur | | | | | | □ | |  | | | | | | |
|  | | | | | | | | | | | | | | |
| 8. | | | Cantitatea de calciu într-un pahar de lapte gras comparativ cu un pahar de lapte degresat este: (bifați o casetă) | | | | | | | | | | | |
| Aproximativ aceeași | | | | | | ✓ | |  | | | | | | |
| Mult mai mult | | | | | | □ | |  | | | | | | |
| Mult mai puțin | | | | | | □ | |  | | | | | | |
| Nu sunt sigur | | | | | | □ | |  | | | | | | |
|  | | | | | | | | | | | | | | |
| 9. | | | Care din următorii nutrienți are cele mai multe calorii pentru aceeași cantitate de aliment? (bifați o casetă) | | | | | | | | | | | |
| Zahăr | | | | | | □ | |  | | | | | | |
| Amidon | | | | | | □ | |  | | | | | | |
| Fibre | | | | | | □ | |  | | | | | | |
| Grăsimi | | | | | | ✓ | |  | | | | | | |
| Nu sunt sigur | | | | | | □ | |  | | | | | | |
|  | | | | | | | | | | | | | | |
| 10. | | | Comparativ cu alimente minim procesate, alimentele procesate au: (bifați o casetă) | | | | | | | | | | | |
| Mai multe calorii | | | | | | ✓ | |  | | | | | | |
| Mai multe fibre | | | | | | □ | |  | | | | | | |
| Mai puțină sare | | | | | | □ | |  | | | | | | |
| Nu sunt sigur | | | | | | □ | |  | | | | | | |

| **Secțiunea 3: Următorii câțiva pași sunt despre alegerea alimentelor** | | | | | | | | |
| --- | --- | --- | --- | --- | --- | --- | --- | --- |
|  | | | | | | | | |
| 1. | | | Dacă o persoană ar vrea să cumpere iaurt de la supermarket, care ar avea cel mai puțin zahăr/îndulcitor?(bifați o casetă) | | | | | |
| Iaurt de cireșe cu 0% grăsime | | | | □ | |  | | |
| Iaurt natural | | | | ✓ | |  | | |
| Iaurt de fructe cremos | | | | □ | |  | | |
| Nu sunt sigur | | | | □ | |  | | |
|  | | |  | | | | | |
| 2. | | | Dacă o persoană ar vrea supă la un restaurant, care ar fi opțiunea cu cantitatea cea mai mică de grăsimi? (bifați o casetă) | | | | | |
| Supă de ciuperci cu risotto (ciuperci de câmp, ciuperci, orez, unt, smântână, pătrunjel și piper negru măcinat) | | | | | | | □ |  |
| Supă de morcovi și dovleac plăcintar iute (morcovi, dovleac plăcintar, cartofi dulci, chimen, ardei iute, semințe de coriandru și lămâie) | | | | | | | ✓ |  |
| Supă cremoasă de pui (pui, ceapă, țelină, cartofi, usturoi, salvie, făină, smântână) | | | | | | | □ |  |
| Nu sunt sigur | | | | | | | □ |  |
|  | | | | | | | | |
| 3. | | | Care ar fi cea mai sănătoasă și echilibrată alegere pentru un fel principal într-un restaurant? (bifați o casetă) | | | | | |
| Friptură de curcan, cartofi piure și legume | | | | | | | ✓ |  |
| Tocăniță de vită, brioșe sărate și cartofi copți | | | | | | | □ |  |
| Pește și cartofi prăjiți servit cu mazăre boabe și sos tartar | | | | | | | □ |  |
| Nu sunt sigur | | | | | | | □ |  |
|  | | | | | | | | |
| 4. | | | Care ar fi cel mai sănătos și echilibrat sandwich pentru prânz? (bifați o casetă) | | | | | |
| Sandwich cu șuncă + fructe + brioșă cu afine + suc de fructe | | | | | | | □ |  |
| Sandwich cu ton+ fructe + iaurt degresat + apă | | | | | | | ✓ |  |
| Sandwich cu salată de ou + o pună mică de chipsuri+ iaurt degresat + apă | | | | | | | □ |  |
| Nu sunt sigur | | | | | | | □ |  |
|  | | | | | | | | |
| 5. | | | Care dintre următoarele alimente ar fi cea mai sănătoasă variantă pentru un desert? (bifați o casetă) | | | | | |
| Sorbet de fructe de pădure | | | | | | | ✓ |  |
| Plăcintă cu mere si mure | | | | | | | □ |  |
| Cheesecake cu lămâie | | | | | | | □ |  |
| Prăjitură cu morcovi și topping de cremă de brânză | | | | | | | □ |  |
| Nu sunt sigur | | | | | | | □ |  |
|  | | | | | | | | |
| 6. | | | Care dintre aceste combinații de legume într-o salată ar oferi cea mai mare varietate de vitamine și antioxidanți? (bifați o casetă) | | | | | |
| Salată verde, ardei verde și varză | | | | □ |  | | | |
| Brocoli, morcovi și roșii | | | | ✓ |  | | | |
| Ardei roșu, roșii și varză | | | | □ |  | | | |
| Nu sunt sigur | | | | □ |  | | | |
|  | | | | | | | | |
| 7. | | Dacă o persoană ar dori să-și reducă cantitatea de grăsimi în dietă, dar nu ar vrea să renunțe la cartofi prăjiți, care dintre aceste variante ar fi cea mai bună opțiune? (bifați o casetă) | | | | | | |
| Cartofi prăjiți tăiați gros | | | | ✓ |  | | | |
| Cartofi prăjiți tăiați subțire | | | | □ |  | | | |
| Cartofi prăjiți tăiați ondulat | | | | □ |  | | | |
| Nu sunt sigur | | | | □ |  | | | |
|  | | | | | | | | |
| 8. | Un mod sănătos de a adăuga gust alimentelor fără a adăuga sare sau grăsimi în plus este a folosi: (bifați o casetă) | | | | | | | |
| Lapte de cocos | | | | □ |  | | | |
| Plante aromate | | | | ✓ |  | | | |
| Sos de soia | | | | □ |  | | | |
| Nu sunt sigur | | | | □ |  | | | |
|  | | | | | | | | |
| 9. | Care dintre următoarele metode de a găti necesită grăsime adăugată? (bifați o casetă) | | | | | | | |
| Gătit la grătar | | | | □ |  | | | |
| Gătit la abur | | | | □ |  | | | |
| Copt | | | | □ |  | | | |
| Soté | | | | ✓ |  | | | |
| Nu sunt sigur | | | | □ |  | | | |
|  |  | | | | | | | |
| 10. | “Alimentele ușoare“ sau light (sau alimentele dietetice) sunt mereu opțiuni bune, deoarece au un conținut redus de calorii.  (bifați o casetă) | | | | | | | |
| Sunt de acord | | | | □ |  | | | |
| Nu sunt de acord | | | | ✓ |  | | | |
| Nu sunt sigur | | | | □ |  | | | |

| Următoarele întrebări sunt legate de etichetele alimentare: | | | | |
| --- | --- | --- | --- | --- |
| \| PRODUSUL 1  BRÂNZĂ TOPITĂ CU ȘUNCĂ  Valori nutriționale la 100g de produs:  Energie – 1148kj / 277 kcal  Lipide 24,6  din care grăsimi saturate 16g  Glucide 5,5g  din care zahăr 5g  Proteine 8,5g  Fibre 0g  Sare 2g \| PRODUSUL 2  BRÂNZĂ TOPITĂ CU SMÂNTÂNĂ  Valori nutriționale la 100g de produs:  Energie 849kj / 205 kcal  Lipide 16,5  din care grăsimi saturate 10,9g  Glucide 5g  din care zahăr 2g  Proteine 9g  Fibre 0g  Sare 2,5g \| \| --- \| --- \| | | | | |
| 11. | | Privind informațiile nutriționale ale produselor 1 și 2, care aliment are cele mai multe calorii (kcal) pe 100g (bifați o casetă) | | |
| Produsul 1 | | | ✓ |  |
| Produsul 2 | | | □ |  |
| Amândouă au aceeași valoare calorică | | | □ |  |
| Nu sunt sigur | | | □ |  |
|  | | | | |
| 12. | Dacă ați dori să alegeți un produs mai sărac în grăsimi, pe care l-ați alege? (bifați o casetă) | | | |
| Produsul 1 | | | □ |  |
| Produsul 2 | | | ✓ |  |
| Nu sunt sigur | | | □ |  |
|  | | |  |  |
| 13. | Dacă ați dori să alegeți un produs mai sărac în zahăr, pe care l-ați alege? (bifați o casetă) | | | |
| Produsul 1 | | | □ |  |
| Produsul 2 | | | ✓ |  |
| Nu sunt sigur | | | □ |  |

| **Secțiunea 4: Această secțiune este despre problemele de sănătate sau bolile legate de dietă și managementul greutății** | | | | | | |
| --- | --- | --- | --- | --- | --- | --- |
|  | | | | | | |
| 1. | | | Care dintre aceste boli este legată de un aport scăzut de fibre? (bifați o casetă) | | | |
| Constipație | | | | ✓ |  | |
| Anemie | | | | □ |  | |
| Carii dentare | | | | □ |  | |
| Nu sunt sigur | | | | □ |  | |
|  | | | | | | |
| 2. | | Care dintre aceste boli este legată de cantitatea de zahăr consumată de populație? (bifați o casetă) | | | | |
| Diabet zaharat | | | | □ |  | |
| Carii dentare | | | | ✓ |  | |
| Anemie | | | | □ |  | |
| Nu sunt sigur | | | | □ |  | |
|  | | | | | | |
| 3. | | Care dintre aceste boli este legată de aportul de sare (sau sodiu)? (bifați o casetă) | | | | |
| Hipotiroidism | | | | □ |  | |
| Diabet | | | | □ |  | |
| Hipertensiune arterială | | | | ✓ |  | |
| Nu sunt sigur | | | | □ |  | |
|  | | | | | | |
| 4. | Care dintre aceste opțiuni este recomandă de experți pentru a reduce șansele de a avea cancer? (bifați o casetă) | | | | | |
| Consumul regulat de alcool | | | | □ |  | |
| Consumul diminuat de mezeluri | | | | ✓ |  | |
| Evitarea zahărului din alimente | | | | □ |  | |
| Nu sunt sigur | | | | □ |  | |
|  | | | | | | |
| 5. | Care dintre aceste opțiuni este recomandată de experți pentru a preveni bolile de inimă? (bifați o casetă) | | | | | |
| Utilizarea suplimentelor nutritive | | | | □ |  | |
| Consumul de pește mai puțin gras | | | | □ |  | |
| Consumul mai redus de grăsimi trans | | | | ✓ |  | |
| Nu sunt sigur | | | | □ |  | |
|  | | | | | | |
| 6. | Care dintre aceste opțiuni este recomandată de experți pentru a preveni diabetul și obezitatea? (bifați o casetă) | | | | | |
| Consumul de alimente mai puțin rafinate/procesate | | | | ✓ |  | |
| Consumul mai mare de suc de fructe | | | | □ |  | |
| Consumul mai mare de carne procesată | | | | □ |  | |
| Nu sunt sigur | | | | □ |  | |
|  | | | | | | |
| 7. | | | Care dintre aceste alimente este mai probabil să crească colesterolul sanguin? (bifați o casetă) | | | |
| Ouă | | | | □ | |  |
| Uleiuri vegetale | | | | □ | |  |
| Grăsimi animale | | | | ✓ | |  |
| Nu sunt sigur | | | | □ | |  |
|  | | | | | | |
| 8. | | | Care dintre aceste alimente este clasificat ca având un indice glicemic ridicat (indice glicemic ridicat înseamnă o creștere mai mare a glucozei în sânge după o masă)? (bifați o casetă) | | | |
| Cereale integrale | | | | □ | |  |
| Pâine albă | | | | ✓ | |  |
| Fructe și legume | | | | □ | |  |
| Nu sunt sigur | | | | □ | |  |
|  | | | | | | |
| 9. | | | Pentru a menține o greutate corporală sănătoasă, ar trebui să eliminată complet grăsimea. (bifați o casetă) | | | |
| Sunt de acord | | | | □ | |  |
| Nu sunt de acord | | | | ✓ | |  |
| Nu sunt sigur | | | | □ | |  |
|  | | | | | | |
| 10. | | | Pentru a menține o greutate corporală sănătoasă, o persoană ar trebui să aibă o dietă foarte bogată în proteine. (bifați o casetă) | | | |
| Sunt de acord | | | | □ | |  |
| Nu sunt de acord | | | | ✓ | |  |
| Nu sunt sigur | | | | □ | |  |
|  | | | | | | |
| 11. | | | Consumul de pâine cauzează întotdeauna creștere în greutate. (bifați o casetă) | | | |
| Sunt de acord | | | | □ | |  |
| Nu sunt de acord | | | | ✓ | |  |
| Nu sunt sigur | | | | □ | |  |
|  | | | | | | |
| 12. | | | Consumul de fibre pot reduce șansele creșterii în greutate. (bifați o casetă) | | | |
| Sunt de acord | | | | ✓ | |  |
| Nu sunt de acord | | | | □ | |  |
| Nu sunt sigur | | | | □ | |  |
|  | | | | | | |

| 13. | Care dintre aceste opțiuni poate ajuta la menținerea unei greutăți corporale sănătoase? (răspundeți la fiecare opțiune) | | | | | | | | | |  |
| --- | --- | --- | --- | --- | --- | --- | --- | --- | --- | --- | --- |
|  | | | | Da | | | Nu | | Nu sunt sigur |  |  |
| Abținerea din consumul de alimente in fața televizorului | | | | ✓ | | | □ | | □ |  |  |
| Citirea etichetelor produselor alimentare | | | | ✓ | | | □ | | □ |  |  |
| Utilizarea suplimentelor nutritive | | | | □ | | | ✓ | | □ |  |  |
| Monitorizarea consumului alimentar | | | | ✓ | | | □ | | □ |  |  |
| Monitorizarea greutății corporale | | | | ✓ | | | □ | | □ |  |  |
| Ciugulit/ ronțăit câte ceva pe tot parcursul zilei | | | | □ | | | ✓ | | □ |  |  |
|  | | | | | | | | | | |  |
| 14. | Dacă cineva are Indicele de Masă Corporal (IMC) de 23kg/m^2^, care ar fi statusul lui de greutate? (bifați o casetă) | | | | | | | | | |  |
| Subponderal | | | | □ | | |  | | | |  |
| Greutate normală | | | | ✓ | | |  | | | |  |
| Supraponderal | | | | □ | | |  | | | |  |
| Obez | | | | □ | | |  | | | |  |
| Nu sunt sigur | | | | □ | | |  | | | |  |
|  | | | | | | | | | | |  |
| 15. | | Dacă cineva are Indicele de Masă Corporal (IMC) de 31 kg/m^2^, care ar fi statusul lui de greutate? (bifați o casetă) | | | | | | | | |  |
| Subponderal | | | | □ | | |  | | | |  |
| Greutate normală | | | | □ | | |  | | | |  |
| Supraponderal | | | | □ | | |  | | | |  |
| Obez | | | | ✓ | | |  | | | |  |
| Nu sunt sigur | | | | □ | | |  | | | |  |
| Priviți aceste forme ale corpului: | | | | | | | | | | |  |
| 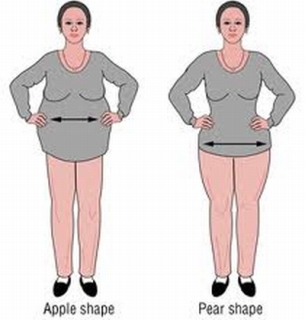 | | | | | | | | | | |  |
| 16. | Care dintre aceste forme ale corpului crește riscul bolilor cardiovasculare (boala cardiovasculară este un termen general care descrie o boală a inimii, a vaselor de sânge, de exemplu, angină pectorală, infarct miocardic , insuficiență cardiacă, boli cardiace congenitale și accident vascular cerebral)? (bifați o casetă) | | | | | | | | | |  |
| Forma de măr | | | | ✓ | | |  | | | |  |
| Forma de pară | | | | □ | | |  | | | |  |
| Nu sunt sigur | | | | □ | | |  | | | |  |
|  | | | | | | | | | | |  |
| **Secțiunea 5: Am dori să vă punem câteva întrebări despre dumneavoastră** | | | | | | | | | | |  |
|  | | | | | | | | | | |  |
| 1. Sunteți… | | | | | | | | | | |  |
| Bărbat | | | | | □ |  | | | | |  |
| Femeie | | | | | □ |  | | | | |  |
|  | | | | | | | | | | |  |
| 2. Care este greutatea dumneavoastră actuală? Vă rugăm să notați kilograme. | | | | | | | | | | |  |
| Kilograme | | |  | | | | |  | | |  |
|  | | | | | | | | | | |  |
| 3. Care este înălțimea dumneavoastră actuală? Vă rugăm să notați în centimetri. | | | | | | | | | | |  |
| Centimetri | | |  | | | | |  | | |  |
|  | | | | | | | | | | |  |
| 4. În general, ați spune că sănătatea dumneavoastră este… | | | | | | | | | | |  |
| Precară | | | | | □ |  | | | | |  |
| Acceptabilă | | | | | □ |  | | | | |  |
| Bună | | | | | □ |  | | | | |  |
| Foarte bună | | | | | □ |  | | | | |  |
| Excelentă | | | | | □ |  | | | | |  |
|  | | | | | | | | | | |  |
| 5. Sunteți… | | | | | | | | | | |  |
| Singur | | | | | □ |  | | | | |  |
| Căsătorit | | | | | □ |  | | | | |  |
| Cu concubin(ă) | | | | | □ |  | | | | |  |
| Separat | | | | | □ |  | | | | |  |
| Divorţat | | | | | □ |  | | | | |  |
| Văduv | | | | | □ |  | | | | |  |
|  | | | | | | | | | | |  |
| 6. Aveți copii?? | | | | | | | | | | |  |
| Nu | | | | | □ |  | | | | |  |
| 1 | | | | | □ |  | | | | |  |
| 2 | | | | | □ |  | | | | |  |
| 3 | | | | | □ |  | | | | |  |
| 4 | | | | | □ |  | | | | |  |
| Mai mult de 4 | | | | | □ |  | | | | |  |
|  | | | | | | | | | | |  |
| 7. Aveți copii minori, care trăiesc cu dumneavoastră? | | | | | | | | | | |  |
| Da | | | | | □ |  | | | | |  |
| Nu | | | | | □ |  | | | | |  |
|  | | | | | | | | | | |  |
|  | | | | | | | | | | |  |
| 8. Care este cel mai înalt nivel de educație pe care l-ați finalizat? | | | | | | | | | | |  |
| Scoala primară | | | | | □ |  | | | | |  |
| Școală gimnazială | | | | | □ |  | | | | |  |
| Școala tehnică | | | | | □ |  | | | | |  |
| Studii liceale fără bacalaureat | | | | | □ |  | | | | |  |
| Studii liceale cu bacalaureat  Studii postliceale | | | | | □  □ |  | | | | |  |
| Studii universitare - licență | | | | | □ |  | | | | |  |
| Studii universitare - master | | | | | □ |  | | | | |  |
| Diplomă postuniversitară | | | | | □ |  | | | | |  |
|  | | | | | | | | | | |  |
|  | | | | | | | | | | |  |
| 10. Aveți vreo calificare în legătură cu nutriția (sau studiați pentru a obține o calificare nutrițională)? | | | | | | | | | | |  |
| Nu | | | | | □ |  | | | | |  |
| Da | | | | | □ |  | | | | |  |
| Vă rugăm, precizați: | | | | | | | | | | |  |
| _______________________________________________ | | | | | | | | | | |  |
|  | | | | | | | | | | |  |
| **Mulțumim pentru participarea în acest sondaj!** | | | | | | | | | | |  |
